# A Hope Intervention Compared to Friendly Visitors as a Technique to Reduce Depression among Older Nursing Home Residents

**DOI:** 10.1155/2010/676351

**Published:** 2010-06-20

**Authors:** Donna M. Wilson, Alexandra Marin, Param Bhardwaj, Bonnie Lichlyter, Amy Thurston, Deepthi Mohankumar

**Affiliations:** ^1^Faculty of Nursing, University of Alberta, Edmonton, AB, Canada T6G 2G3; ^2^Department of Family Medicine, University of Alberta, AB, Canada T6G 2C8

## Abstract

Depression is common among older persons. An experimental study was undertaken to test the impact of a four-week hope program on depressed nursing home residents. Residents aged 65 or older, who met the criteria for this pilot study and agreed to participate, were randomly assigned to (a) an intervention group, and provided with weekday hope interventions mainly involving positive messages and pictures or (b) a modified control group, and provided with a friendly weekday greeting. The structured hope intervention was not proven effective for reducing depression or raising hope. Instead, a significant reduction in depression among the control subjects was found, as well as a nonsignificant increase in their level of hope. Although these findings suggest friendly visitors may be a more efficacious nonpharmacological approach for reducing depression, further investigations are needed to confirm this and to explore the impact of other hope interventions.

## 1. Introduction

Depression is a significant health problem affecting 14.7% to 30% of all older persons [1, 2]. Depression rates are typically higher among senior citizens who live in nursing homes, as compared to those who live in community settings [3–5]. Between 30% and 40% of older persons living in nursing homes and other institutional settings have been reported as suffering from some degree of depression [6]. Depression is often simply thought of as a constellation of negative feelings such as hopelessness, sadness, anger, and irritability. Depression is much more than an emotional response, however, as depression affects behavior and both cognitive and physical health [7]. Depression is now clearly linked with an increased susceptibility for acquiring other serious health problems [8, 9], and with a higher risk of death from suicide and other causes [10]. 

In spite of the common perception that depression is a natural or perhaps even inevitable aspect of the aging process [11], depression among older persons is not normal and it should be treated as an illness that can be cured or at least moderated [12]. Typical interventions for depression are psychotherapy and pharmaceuticals, or a combination of the two [13]. Unfortunately, research is demonstrating that drug therapy outcomes are not always optimal for older persons and that nonpharmacological treatments should be increasingly considered [1, 9]. Some studies have demonstrated the benefits of alternative therapies such as exercise, light therapy, and herbs [14]. The experimental study presented here tested the effects of a four-week hope program for depressed older persons living in a large nursing home in the western Canadian province of Alberta. This small pilot study primarily involved a between and within groups comparison of data collected through the Geriatric Depression Scale Short Form and the Herth Hope Index.

## 2. Literature Review

Older persons who live in institutions such as nursing homes are highly vulnerable to depression, in part because they have become isolated from familiar surroundings, neighbors, and family members [15, 16]. An episode of severe illness, a decline in health, and the passing of a spouse often precedes their admission to a care facility, with this transition in health, wellbeing, family connections, and/or living arrangements contributing to depression. Depression may have also been present earlier in the person's life, with episodes of moderate to severe depression potentially necessitating treatment. A decline in personal strength is another possible factor for depression among nursing home residents, with this decline thought by some to be an outcome of aging and the many losses that can accompany old age [17]. Clearly, given the high rate of depression among institutionalized older persons, efforts to reduce depression are important to explore. 

The therapeutic value of hope to health, healing, wellbeing, and quality of life is now well documented [18]. In the last three decades, interest in hope has intensified, as shown by a surge in qualitative and quantitative studies—many of which having occurred in healthcare settings. To date, this body of research has linked hope and healing [19], hope and coping [20–22], hope and goal setting [23], hope and finding meaning in suffering and illness [2, 24, 25], and hope as an antidote to hopelessness [26]. Among healthcare professionals, hope has been identified as a fundamental necessity [20], being vital to both life and living [25], and an essential human need—particularly among elderly persons [27]. Research has emphasized the relational nature of hope, exploring in particular the centrality of caregivers, family, and friends to fostering hope [28]. Nurses have also been found to be in a unique position to offer hope to their patients or clients [18, 24, 29, 30].

Spirituality and faith have been identified as additional factors influencing hope [24, 26]. Hope is more often considered a crucial component of spiritual care. Religious beliefs, including faith in a higher power, life after death, and the power of prayer, can raise hope. Davis's [31] study involving people aged 60–89 confirmed positive correlations between hope and spirituality, hope and well-being, and spirituality and well-being. The research to date on hope has thus revealed it as a life force, something that individuals can benefit from in times of crisis, tragedy, or illness.

Exploring the potential for hope to be instilled, nurtured, and/or encouraged among depressed older persons living in nursing homes is perhaps one of the most important steps now for researching the therapeutic value of hope. Several studies have already explored levels of hope among older persons [21, 30, 32, 33]. Others have studied hope among persons with specific afflictions such as cancer [34, 35] and stroke [36]. Few studies, however, have explored hope as an intervention. One such study used the Herth Hope Index (HHI) as a measurement tool in a pre-post test design involving two hope interventions (a 20-minute hope video and a daily reflective journal) for persons receiving or providing palliative care [34]. No studies on hope as an intervention for depressed older persons in nursing homes or other institutional care settings appear to have been conducted.

## 3. Research Methodology

A pilot study was planned and conducted in one Canadian nursing home to discriminate the effects of a four-week hope program on depression and hope. This nursing home was convenient to the researchers, and with a one-site study planned to control for intervening effects that are likely to occur with multiple study sites. A single study site was also considered an ethical option, as the hope program had been newly developed by a multidisciplinary facility team and its effects are untested. This hope intervention program was designed after the hope literature had been reviewed and local hope experts consulted. An experimental study was designed involving a randomized-controlled trial design.

Two established measurement tools, the Hearth Hope Index (HHI) and the Geriatric Depression Scale Short Form (GDSSF), were selected to gather comparative data among subjects. The HHI, a shorter (12-item) version of the original Hearth Hope Scale, was designed specifically for use in clinical settings [37]. The HHI employs a four-point Likert scale per item to measure dimensions of hope, with total potential tool scores ranging from 12 to 48. This tool is easy to understand and quick to administer, with the developer indicating it is as effective as the original longer Herth Hope Scale [37]. Construct validity, internal consistency, and test-retest correlations have continued to establish the efficacy of both Herth Hope tools.

The GDSSF is a 15-item tool that was developed as a short form of the original Geriatric Depression Scale Long Form, with this shorter tool also identified as valid and reliable [38, 39]. The GDSSF is easy to understand and quick to administer in clinical settings [38], with this tool specifically chosen for this study because the target population was depressed older persons living in a nursing home. This study was approved by the administrators of the nursing home and also by the University of Alberta's Health Research Ethics Board, after one major change to the experimental design was made as mandated. This change was that the persons randomly assigned to the control group would receive a brief friendly weekday greeting from the Research Assistant instead of their only receiving their usual daily care. This study involved four research phases.

### 3.1. Phase One: Participant Screening, Selection, and Securing of Informed Consent

The nursing home where the study was conducted has 436 continuing care beds and thus 436 potential subjects, as 100% bed occupancy is common. The inclusion criteria for this study, however, were that all subjects had to be 65 years of age or older, diagnosed with and continuing to suffer from depression, able to voluntarily sign an informed consent form, could speak and understand English, had resided in this nursing home for three or more months, had no changes in their depression drug therapy in the three months prior to the initiation of this study, and also no changes in their depression drug therapy throughout the four-week study. A total of 58 potential subjects were identified by the facility's nursing managers as meeting all inclusion criteria. 

The charts of these 58 persons were reviewed for inclusion criteria, with all 58 remaining as potential research subjects. A flyer describing the study was then circulated to these 58 residents, with a member of the research team approaching each later that week to inform them about the study. Each potential subject, who agreed to be involved in the study, was then tested for mental competency using the MDS Cognitive Performance Scale (CPS), a tool often administered in nursing homes to determine mental competency [40]. This test is highly correlated with the Mini-Mental Status Examination [41]. All persons who scored 2 or less were retained in the potential subject pool, as a CPS score of 2 or less indicates mild or no cognitive impairment. This testing was done to ensure each person was cognitively able to provide informed consent. 

The goal for this experimental study was to recruit at least 40 subjects, with 20 randomized to the control group and 20 the intervention or treatment group, in order to achieve statistically meaningful findings. However, among the 58 possible subjects, 8 lacked cognitive capacity to make decisions and 1 refused to take the CPS test; these 9 were excluded from the study. Of the remaining 49, 19 were not currently depressed as they were found to have a score of 3 or more on the Depression Rating Scale (a tool developed and routinely used in this nursing home to identify clinically depressed residents). Another 13 were excluded due to low comprehension of the English language or lack of interest in the study. The remaining 17 participants were randomly assigned to the control group or the intervention group, with 9 becoming controls and 8 intervention group members. The 8 persons who were randomly assigned to the treatment group received a brief weekday hope intervention from a Research Assistant over four consecutive weeks. The 9 persons randomly assigned to the control group received a brief friendly weekday greeting from the Research Assistant over the same time period.

### 3.2. Phase Two: Gathering Baseline HHI and GDSSF Data, and sociodemographic Data

Immediately prior to the study, all 17 subjects were administered the GDSSF and HHI tests to gather baseline hope and depression data. In addition, select sociodemographic data were collected from each chart to enable a description of the participants and to permit a comparison of control and treatment subjects. The Research Assistant also began to keep field notes to record her perceptions of the effect of her greeting on control subjects and the effect of the various hope interventions on treatment subjects.

### 3.3. Phase Three: Four-Week Study Period

Over the four weeks, the control and intervention group subjects continued to receive their usual prescribed medications and all care normally provided to them. As indicated above, each subject in the control group was also briefly visited each weekday by the Research Assistant, a daily visit mandated by the Health Research Ethics Board to ensure their ongoing willingness and ability to continue in the study, and to prevent them from feeling neglected while taking part in a research study that provided other residents in the nursing home with visible hope interventions. 

Subjects who received the hope intervention were also visited each weekday over the same four-week period, although the focus of each visit was to provide them with that day's hope intervention. During the first week, a “hope card” was delivered each weekday. This card contained an inspirational message in large print. The message was read to the participant and the card was left for them to keep. Over the second week, in addition to receiving a new hope card each weekday, the participant was asked to recall a time in the past when they had experienced hope and to share this experience with the Research Assistant. In the third week, different hope pictures were shown to each subject every weekday. These pictures had an accompanying message that was read to the subject by the Research Assistant. The subject was then asked to set a goal for the day. The Research Assistant added this goal to a card that had been prepared in advance with the words “Today, my goal is…” This card was left for the subject to see and read. In addition, in week three, a short qualitative interview not lasting more than 30 minutes took place. This interview was designed to gain information associated with two questions: (a) can you recall a time when you had hope? and (b) would you please tell me about that time? In the fourth and final week, each subject was given a choice of one picture or image that represented hope from among four presented to them each weekday. Participants were asked to say why the chosen picture or image represented hope to them. This picture was then left with the participant.

### 3.4. Phase Four: Final Data Collections and Comparisons

At the end of the four-week period, the HHI and GDSSF were readministered to the 15 remaining participants, with two subjects in the intervention group having dropped out of the study by then. The data from the two tools collected at both intervals (pre- and poststudy) and the sociodemographic data were entered for all 15 subjects into an SPSS spreadsheet. The hope and depression data were compared between and within groups using *t*-tests, a test appropriate for the data variables and the specific purpose of this experimental study to test the efficacy of the hope intervention on hope and depression. The qualitative data recorded over the course of this study by the Research Assistant were also reviewed by the research team. This review of the field notes was mainly undertaken to help the research team understand the findings.

## 4. Findings

As indicated previously, 9 subjects were initially randomized to the control group and 8 to the intervention group. The two participants in the intervention group who dropped out did so because of a negative reaction to the hope interventions. One indicated that she was too tired to take part in the study, as it “took too much out of her,” and the other said she did not like the study. No subjects in the control group dropped out, and no negative reactions to this study among these subjects were noted by the Research Assistant. Instead, consistent positive reactions among the control subjects were noted, with some openly stating that they looked forward to the brief weekday visit from the Research Assistant. 

Table 1 outlines the sociodemographic findings for the two groups. These findings represent the 15 subjects who completed this study, as replacements for the two subjects who withdrew were not possible. The average age, average length of nursing home stay, average Cognitive Performance Scale (CPS) score, and average Depression Rating Scale (DRS) score did not differ significantly between the control and intervention subjects. As such, the control and intervention group subjects were relatively similar in age, length of stay in the nursing home, cognitive ability, and degree of depression. Table 1 also outlines the average pre- and poststudy GDSSF and HHI scores for the control group and the treatment group. No difference in the average prestudy GDSSF or the average prestudy HHI scores between the control and intervention groups was found, findings that indicate that these two groups were initially quite similar to each other with regard to their degree of depression and their level of hope. 

Table 1 also shows that the average poststudy GDSSF and HHI scores for both the control and intervention group subjects continued to be similar to each other after the four-week study ended. These findings indicate that there was no difference in the effect of the four-week hope intervention program as compared to the brief informal visits by the Research Assistant. 

Table 2 outlines the average pre- and poststudy GDSSF and HHI scores for the control group and the average pre- and poststudy GDSSF and HHI scores for the treatment group. After these scores were compared within groups, one significant difference was found. The intervention group had a statistically significant drop in their average hope scores (from 33.0 to 32.0), a finding that indicates their level of hope declined despite their having received a month-long hope intervention program. In contrast, the measured level of hope among the control subjects increased, although this increase was not statistically significant. Another key finding was that the depression scores among the control group subjects declined to a near significant level, while the intervention group's depression scores declined only slightly, indicating that the control subjects had improved more by having less depression as compared to the intervention subjects.

## 5. Discussion

As indicated, the purpose of this experimental study was to test the efficacy of a relatively simple and inexpensive month-long hope intervention program for depressed older persons living in a nursing home, with the ultimate goal of this study to gain research evidence relevant to alleviating or reducing depression among nursing home residents through nonpharmacological methods. Psychotherapeutic and pharmacotherapy are common approaches to treating depression, while hope treatments for depression (such as the one used in this study) have not previously been examined for their effects. The findings of this pilot study are both surprising and revealing. 

Although it was anticipated that the hope intervention program would relieve depression and improve hope among the treatment group subjects, subjects in the control group appeared to benefit more through their involvement in this study. The control group had a nearly significant decrease in depression while the treatment group only showed a slight decline in depression. As such, the hope intervention was not proven effective for reducing depression. This finding could be a result of sampling or testing issues, with other hope interventions perhaps more helpful for reducing depression. It is important to note, however, that the participants in both groups were not as depressed after the four weeks, a finding that suggests depression among nursing home residents is potentially reducible through nonpharmacological interventions. 

Another important finding was the unanticipated reduction in hope among the treatment group subjects, particularly as compared to the control group where an increase in hope was found. Although the small number of intervention and control group subjects could explain this finding, it is also possible that this specific hope intervention program was not designed appropriately for depressed older persons. Other hope interventions could perhaps have more efficacy for improving hope and reducing depression among nursing home residents. Another possible rationale for the lack of expected treatment effect could be that 30 days is not enough time to instill hope or significantly reduce depression among nursing home residents. Nursing home residents, who are advanced in age and dependency requiring nursing home level care, may not be as responsive to this or other therapies particularly if they have suffered from long-standing depression. 

Another possible explanation for the unexpected findings of this study is that the subjects in the control group responded more positively to the friendly weekday greeting by the Research Assistant as compared to the less personal and more structured or purposive weekday visit. A growing body of literature is revealing the importance of visitors to help people recover from illnesses, with other positive outcomes also having been identified to date [42]. As such, the unexpected findings of this pilot study suggest that paid or volunteer visitors could potentially relieve depression among institutionalized older persons. Furthermore, these findings suggest care staff should be educated to be outwardly friendly when approaching and while working with depressed older persons. The use of humor by caregivers or visitors could also be considered. Humor is important for building therapeutic relationships and for other therapeutic effects among ill persons [43].

## 6. Conclusion

Given the high rate of depression among older persons, particularly those living in nursing homes, much more should be done to alleviate it. Although this study found that a simple cheerful greeting was more effective for relieving depression and instilling hope than a four-week program of carefully planned and constructed hope interventions, more research is needed to substantiate or refute this conclusion. The surprising yet revealing findings of this study are not generalizable as they are limited by the small sample size, but they provide a starting point for other studies. Studies with a standard control group, larger samples, and multiple nursing homes are encouraged to test the efficacy of hope interventions. Other experimental studies should test the effect of brief social visits for relieving depression in nursing homes. Qualitative or mixed-methods studies are also encouraged to gain needed insight into nonpharmacological ways of relieving depression.

## Figures and Tables

**Table 1:** Scores of control and intervention groups, with between-group comparisons.

Measures	Control	Intervention	Comparison Test
Number of Subjects	9	6	—
Male	*2*	0	—
Female	7	6	—
Mean Age of Patients	82.2	79.7	*T* = .231, df = 13, *P* = .820
Mean Length of Stay (days)	1,099.0	1,203.8	*T* = .573, df = 13, *P* = .575
Mean Cognitive Performance Scale (CPS) Score	1.22	1.17	*T* = .24, df = 13, *P* = .814
Mean Depression Rating Scale (DRS) Score	0.78	1.67	*T* = 1.134, df = 13, *P* = .274
Mean Prestudy GDSSF	9.33	9.33	*T* = 0.000, df = 13, *P* = 1.00
Mean Poststudy GDSSF	7.11	7.17	*T* = 0.36, df = 13, *P* = .972
Mean Prestudy HHI	31.00	33.00	*T* = 1.125, df = 13, *P* = .281
Mean Poststudy HHI	32.33	32.00	*T* = 0.219, df = 13, *P* = .830

**Table 2:** Scores of control and intervention groups, and within-group comparisons.

Measures	Mean PreStudy Score (SD)	Mean PostStudy Score (SD)	Statistical Test
Control GDSSF	9.33 (1.76)	7.11 (2.80)	*T* = 2.294, df = 8, *P* = .051
Intervention GDSSF	9.33 (1.72)	7.17 (2.46)	*T* = 1.060, df = 8, *P* = .320
Control HHI	31.00 (3.41)	32.33 (3.78)	*T* = 1.337, df = 5, *P* = .239
Intervention HHI	33.00 (1.43)	32.00 (1.48)	*T* = 2.739, df = 5, *P* = .04*

*Test reveals a significant difference in scores. SD is Standard Deviation.
